# Imported leishmaniasis cases in Cuba (2006–2016): what have we learned

**DOI:** 10.1186/s40794-018-0067-3

**Published:** 2018-08-07

**Authors:** Ana M. Montalvo, Jorge Fraga, Orestes Blanco, Daniel González, Lianet Monzote, Lynn Soong, Virginia Capó

**Affiliations:** 1 Institute of Tropical Medicine “Pedro Kourí”, Autopista Novia del Mediodía Km 6 y ½, Lisa, La Habana, Cuba; 2 Department of Microbiology and Immunology, Department of Pathology, University of Texas Medical Branch (UTMB), Galveston, TX USA

**Keywords:** Leishmaniasis, Diagnosis, Epidemiology, Cuba, Travel medicine

## Abstract

**Background:**

Leishmaniasis is a neglected parasitic disease caused by *Leishmania* spp., which is not endemic in Cuba. However, several factors (such as human activities, climate changes, and tourism) have led to an increase in the number of leishmaniasis cases in all regions, raising diagnosis and surveillance issues. We aim to present the retrospective analysis of 16 human cases suspicious of leishmaniasis, which were received during 2006–2016 for diagnosis at the Department of Parasitology from the Institute of Tropical Medicine Pedro Kourí, Cuba.

**Methods:**

Clinical samples were collected and analyzed via different diagnostic assays, including direct smear, cultivation, histological analysis, and molecular analysis. Epidemiology and background of infection, clinical features, sex and age from each patient was recorded.

**Results:**

From the 16 suspicious cases, 5 cases were confirmed for *Leishmania* infection, based on at least two positive results using different methods: PCR-based diagnosis [18S rRNA (5/5), *hsp*20 gene (4/5), *hsp*70 gene (3/5)], histopathology evaluation (2/3), parasite cultivation (2/3), or direct smears (2/3). *L. braziliensis and L. mexicana* were identified as the involving species in two cases, according to *hsp*70 PCR-RFLP protocols. Demographic and clinical features, as well as treatment and follow up, are described for every case.

**Conclusions:**

The combination of parasitological and molecular methods allowed proper diagnosis of imported leishmaniasis cases in Cuba. The utility and advantages of molecular diagnosis assays in non-endemic countries like Cuba are discussed.

## Background

Over 20 species of *Leishmania* are associated with leishmaniasis in humans, but leishmanial infection is not endemic in Cuba. Parasite-carrying phlebotomine sandflies can infect humans exposed to ecosystems [1], leading to diverse clinical outcomes, depending on multifactorial parameters such as host response or susceptibility and parasite genetic background [2].

Recent statistics indicates that leishmaniasis are endemic in 98 countries (72 are developing nations; and 13 are the least developed ones) [3]. Non-endemic areas such as Cuba, can be also affected due to the high human global mobility, which has increased in recent years. Adventure tours and ecotourism [4, 5], civil cooperation and military conflicts [6, 7] among others, can bring people to close contact with usual environments for vectors and reservoirs of *Leishmania*, favoring their infection.

Different diagnostic methods with a variable sensitivity are available: the direct microscopical observation in smears or histological detection of *Leishmania* amastigotes in tissue samples, in vitro culture for growth of promastigotes, and molecular detection of parasite’s DNA by using PCR techniques. The molecular approach is now the preferred one to use for returning travellers because it is highly sensitive and allow the species identification [8, 9]. The kinetoplast, extrachromosal DNA (kDNA), the ribosomal internal transcribed spacer (ITS), and the 18S fragment from ribosomal RNA [10, 11] (rRNA) are some of the most used targets for *Leishmania* genus detection. A gene fragment from the small (20 kDa) heat shock protein (Hsp20) has also been used for the same purpose [12]. For additional identification of the infecting species, the gene coding for heat shock protein (*hsp*70) has demonstrated to be a valuable target for typing parasites from the New and Old World countries [13–15].

The Institute of Tropical Medicine Pedro Kourí (IPK) is the referral center in Cuba for diagnosis and treatment of infectious diseases, comprising those caused by parasites. When suspicious cases of leishmaniasis arrive to the country, they are attended, diagnosed, and treated. The updating of tools for diagnostic purposes has been a task for the past years. Recently, the presence of Cuban civil personnel enrolled in collaborative duties, the arrival of students from endemic countries to spend long periods of time, as well as a comprehensive increase of personal travelling, has motivated the implementation of molecular methods to support the diagnostic capacity of the laboratory, as recommended for non-endemic areas [16]. These procedures together with the classical parasitological assays (parasite observation by direct exam, histology and culture) form part of the algorithm used presently in the laboratory to provide a proper diagnosis [17].

Although some imported leishmaniasis cases have been diagnosed in Cuba previously [18], there are no detailed reports. Here, we present a retrospective study of available data collected from 16 patients with suspected leishmaniasis that were investigated in our laboratory from 2006 to 2016. The epidemiological, clinical, and diagnostic issues concerning these cases are presented and discussed. Our findings will shed light on imported leishmaniasis in Cuba.

## Methods

### Ethics

The conditions established by the institutional ethical committee (CEI-IPK) were followed during the diagnostic process. All rights and expected benefits were explained to the patients. These include the proper use of their clinical samples to test and investigate different diagnostic procedures, and to receive an adequate treatment and medical follow-up, according to the final diagnosis. The results of laboratory tests were informed in each case by medical doctors in charge.

### Clinical evaluation

Clinical examination of each patient was made at the admission to the Institute of Tropical Medicine “Pedro Kourí”. Epidemiological data were collected and completed from the hospital clinical records (Table 1). A second clinical reevaluation was performed to individuals with leishmaniasis 2–3 months after receiving the treatment, if they remained in the country.

**Table 1:** General data of patients attended at IPK (2006–2016) for leishmaniasis diagnosis purpose

Case number	Country of exposure Date	Patient Sex/Age	Epidemiological background	Clinical features
Case-1	Colombia 2006	M/43	Foreigner. Frequent travels to rural areas in his country.	Unique typical lesion on the right leg. Elevated and well defined borders, dry lesion, non-ulcerated. CL suspicious.
Case-2	Costa Rica 2006	M/23	Foreign student in Cuba. Spent holidays in rural areas of Costa Rica two months prior to the appearance of the first lesion.	Three lesions on legs, one in the left arm. Border well defined, slightly ulcerated. Over infected. CL suspicious.
Case-3	Venezuela 2007	M/41	Cuban civil collaborator working in a periurban area.	Erythematous plaques located near the ankles, in the 6 months prior to admission, and more recently, in the thigh. Diagnosed previously as leishmaniasis at the site of origin. Submitted for confirmation.
Case-4	Bolivia 2009	M/24	Foreign student in Cuba. Used to live in a rural area before arriving at Cuba, 6 months earlier.	Cutaneous lesions on the external edge of the right arm and forearm. Borders defined, ulcerated. Several lesions already cured. Small new ones appearing. Ganglionar chain inflammation. CL suspicious.
Case-5	Venezuela 2009	M/39	Cuban civil collaborator working in a rural community.	Lesions on the nose, obstructive, crusty, over infected and exudative. High inflammation during 2 months of evolution. Differential diagnosis of mucosal leishmaniasis.
Case-6	Angola 2011	M/52	Cuban civil collaborator working in close contact with a variety of vectors.	Hepato and splenomegaly, weakness, high fever. Differential diagnosis of visceral leishmaniasis.
Case-7	Venezuela 2011	F/42	Cuban civil collaborator working in a rural community.	Unique long-lasting lesion on the cheek, no borders, no crusty, no infected. Differential diagnosis of CL.
Case-8	Venezuela 2012	M/48	Cuban civil collaborator working in several rural areas.	Previous lesions resembling small furuncles in legs and thigh. Hypochromic, round lesions in both inferior members. Small vesicles. Differential diagnosis of CL.
Case-9	Brazil 2012	M/45	Cuban civil collaborator working in rural area.	Cutaneous dry lesions on the legs. No borders defined. Some de-pigmented scars. Differential diagnosis of CL.
Case-10	Haiti 2012	F/50	Cuban civil collaborator working in different rural areas.	Long lasting lesion on left leg. No borders defined ulcer or crust. Sometimes itching. Differential diagnosis of CL.
Case-11	Venezuela 2012	F/41	Cuban civil collaborator working in rural areas, comprising some periods in the forest.	Five lesions on the face with defined, indurated borders, a few months of evolution. Some over infected. Ulcers. CL suspicious.
Case-12	Peru 2013	M/52	Foreigner. Frequent traveler to Latin American countries including rural areas.	Disseminated, round and dry lesions along the inferior part of legs, abdomen and torax. Non-ulcerated. Differential diagnosis of CL.
Case-13	Equatorial Guinee 2014	M/22	Foreign student. Used to live in a rural area.	Hepato-splenomegaly. Splenomegaly very pronounced. Pain in all the abdominal left side. Differential diagnosis of visceral leishmaniasis.
Case-14	Venezuela 2014	F/51	Cuban civil collaborator working in several areas including rural and remote ones.	Lesion on the upper part of the back. Borders defined, no crust, slightly ulcerated. No over infection. Previously diagnosed at the site of origin. Confirmation of diagnosis.
Case-15	Bolivia 2014	F/40	Cuban civil collaborator working in rural areas.	Nodular lesion on the left leg, erythematous, crusty. Then appeared purplish, squamous. Small subcutaneous nodules around the lesion. Differential diagnosis of CL.
Case-16	Burundi 2016	F/23	Foreign student. Used to Live in a peri-urban area with occasional travels to rural ones.	Chronic lesions in the legs, two in the arms. Crusty, slightly exudatives and ulcerated. The patient referred to suffer from these lesions long time ago. Differential diagnosis.

### Samples

Lesion scrapings (with sterile lancets), cotton swabs (for the mucosal lesion), or biopsies (disposable punch) were taken indistinctly from the edge of suspected lesions (taking into account their location, time of evolution and clinical condition) and were examined for amastigote and cultured for parasites. Peripheral blood was obtained by venous tap when visceral leishmaniasis was suspected. Two blocks of formalin-fixed embedded skin tissue were also received for analysis. In most cases, a fresh portion of the clinical samples was used for DNA extraction and preservation (− 20 °C), allowing further molecular analyses.

### Microscopic examination

Direct examination of tissue lesion smears were fixed with methanol, stained with Giemsa and analyzed by optical microscopy (1000X magnification). The search of amastigotes was made in different samples, including paraffin-embedded tissues. It was not done in blood (Case-6, Case-13).

### Culture

Lesion scrapings or tissue samples from the peripheral edges of the lesions were taken with a sterile lancet and gently placed in a tube containing biphasic NNN medium (Novy, Mc Neal, Nicolle) by using Schneider’s medium as the liquid phase. The tubes (2 per case) were incubated at 27 °C for promastigote cultures, checking weekly for up to 2 months. Bacterial or fungal contaminated cultures were discarded.

### Histological study

Histopathological method for formalin-fixed paraffin-embedded tissues was routinely used to produce 4-μm thick tissue sections, as described elsewhere [19]. Tissue sections were stained with the Harris hematoxilin and eosine or with the PAS reaction (periodic acid of Schiff) [19]. Slides were read under an optical microscope (Olympus) using 10X, 20X, 40X 100X magnification plus 10X for the optical lens.

### DNA extraction

DNA from lesion biopsies, scrapings, and blood was obtained using QIAamp DNA Mini Kit from Qiagen (Hilden, Germany), according to the manufacturer’s instructions.

When paraffin-embedded tissue were used for DNA extraction, three 10 μm-thick sections were deparaffinized with xylene, washed in ethanol, and digested with proteinase K (Roche Diagnostics GmbH, Mannheim, Germany). The Qiagene QIAmp protocol was followed to extract DNA from these sections. The DNA obtained was checked for integrity on 0.8% agarose gel electrophoresis.

### PCR-18S

A 115-bp sequence within the 18 s rRNA gene of *Leishmania* was used for molecular detection of parasites, by using the primers and thermocycler program previously reported [11]. The 25-μL reaction mixture contained: 1X PCR buffer, 2.5 mM MgCl_2_, 0.8 μM of each primer, 200 μM of each deoxynucleoside triphosphate, 0.1 mg/mL of acetylated bovine serum albumin (Promega, Madison, USA), 0.5 U HotStarTaq DNA Plus polymerase (Qiagen). As template, 5 μL of DNA extracted from lesions or paraffin-embedded biopsies, respectively, was added. Assay controls included negative controls (omitting template DNA) and positive controls, replacing template with 100 fg (5 μL) of DNA from *L. donovani* reference strain MHOM/SD/−−/1S (kindly donated by the Institute of Tropical Medicine of Antwerp, Belgium). To exclude possible presence of inhibition factors in testing samples, we included an additional control, in which 100 fg (1 μL) of DNA from the reference strain was added to a tube mixture containing 4 μL of the DNA from the patient’s sample. After amplification, 20 μL of the PCR products were analyzed on a 2% agarose gel. The protocol was always repeated twice.

### PCR-*hsp*20

For the amplification of a 370-pb fragment of the gene coding for the small hsp of 20 kDa, the primers and conditions reported elsewhere were used [12]. The reaction mixture (25 μL) contained 1× PCR buffer, 2.5 mM MgCl_2_; 1× Q buffer; 200 μM of each deoxynucleoside triphosphate, 0.5 U of HotStarTaq DNA Plus polymerase (Qiagen) and 0.8 μM of each primer. As template, 5 μL of the DNA extracted from clinical samples were added. Proper control groups (described above) were also included. The amplified products (15 μL) were analyzed on a 2% agarose gel; detection of band at the expected size was considered positive. The protocol was repeated twice.

### PCR-*hsp*70

For species detection and further identification, three PCR protocols (*hsp*70-PCR, PCR-F and PCR-N) were assayed indistinctly using the primers and conditions previously reported [13, 14]. When PCR signal was strong enough, restrictions were carried out. Restriction Fragment Length Polymorphism (RFLP) was performed, following the stepwise algorithm described previously [13–15].

Examination of the smear, culture, and histopathological sections was performed in parallel whenever possible. In addition, a portion of the clinical samples was stored, the DNA obtained and preserved at − 20 °C for further confirmation. PCR protocols were assayed retrospectively as they were introduced in the laboratory (Cases 1–10). The analyses of Cases 11–16 was performed at the same time by using different diagnostic assays. Appropriate treatment was indicated to each patient, according to the final diagnosis. For treating leishmaniasis different schemes of the recommended drugs (Pentavalent antimonials and Amphotericin B) were used, depending on their availability. Patients were hospitalized and supervised while receiving the drugs.

## Results

Over a 10 years period (2006–2016), our laboratory investigated samples from 16 cases suspected of leishmaniasis, of which cutaneous leishmaniasis (CL) was confirmed in 5 cases. Among examined cases (10 males; 6 females), 4 were foreign students, 1 was foreign traveler from South America, and 11 were Cuban oversea travelers. In most of cases, epidemiological background supported the possible leishmaniasis infection (Table 1).

According to the information analyzed, several subjects (Cases 1, 2, 4, and 11) had lesion(s) or symptoms clinically compatible with CL and were considered with presumptive diagnosis of leishmaniasis. For Cases 5, 6, 7, 8, 9, 10, 12, 13, 15, and 16, a differential diagnosis of leishmaniasis was requested, among which two subjects (Case 3 and 14) were sent for confirmation before treatment, as they corresponded to patients previously diagnosed with CL abroad. Epidemiological and clinical background is shown in Table 1.

The observation of amastigotes by direct smear was possible in Case 1 and Case 14, while this assay was negative in other 8 cases. Histopathology informed *Leishmania* in Cases 2, 4, and 11. While cultures were established whenever possible, only two samples (Cases 1 and 4) gave positive culture results.

PCR-18 s was used to detect *Leishmania* DNA, and positive amplification results were obtained in 5 samples (Cases 1, 2, 4, 11, and 14). Using PCR-*hsp*20, Cases 1, 2, 4, and 11 were positive as well, but Case 14 was not done. The results obtained from PCR-18S and PCR-*hsp*20 were concordant in every patient for both duplicates.

**Fig. 1:**
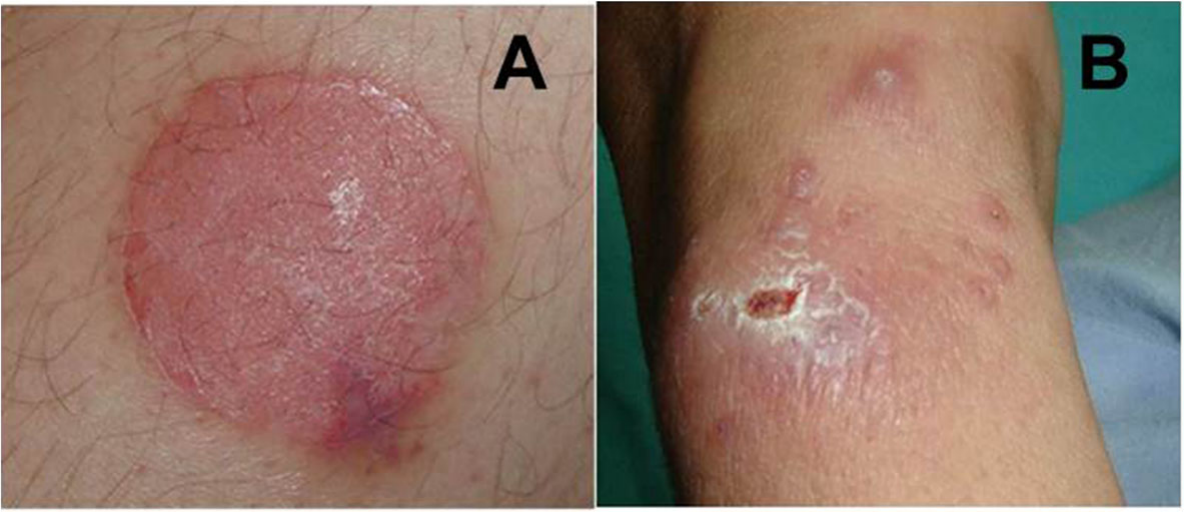
Photographic composition showing lesions from two of the cases analyzed. **a**: An erythematous plate in the leg of the patient corresponding to Case 3, for which the final diagnosis was psoriasis. **b**: Several lesions in different stages of the evolution, from the patient identified as Case 4, diagnosed as cutaneous leishmaniasis

Regarding the cases requested for confirmation, Case 3 was the one that could not confirm leishmaniasis in any of the applied methods (Fig. 1), even after two repeats of sampling and diagnostic procedures within a three-week interval. Case 3 was previously diagnosed as CL, according to the clinical appearance and histological examination. On the other hand, Case 14 was confirmed as CL by direct smear examination (as it was diagnosed in the country of exposure) and PCR-18S as well. The results of our methods used and cases with positive diagnosis of leishmaniasis are presented in Table 2. Figure 1 is a graphic composition, showing images from Case 3 (previously diagnosed for leishmaniasis, but negative in our assays) and Case 4 (suspicious and confirmed as leishmaniasis), respectively.

**Table 2:** Results of diagnostic tests and treatment provided to leishmaniasis cases

Case number	Occupation	Sample	Direct smear	Culture	Histopathology	PCR-18S	PCR-hsp20	PCR-hsp70	Leishmania spp. identified	Treatment regime
Case-1	Not known	Biopsy	Positive	Positive	Not done	Positive	Positive	Positive	*L.*(*V.*) *braziliensis*	Glucantime (20 mg/Kg/day) for 20 days*
Case-2	Student	Biopsy	Not done	Contaminated	Elements in macrophages compatible with *Leishmania* amastigotes	Positive	Positive	Positive	*L.* (*L.*) *mexicana*	Amphotericin B 25 mg × 3 days 50 mg × 12 days
Case-4	Student	Biopsy	Negative	Positive contaminated	Elements similar to amastigotes in macrophages. No kinetoplast identified	Positive	Positive	Positive weak	No identified	Liposomal Amphotericin B 150 mg days 1–5 followed by 200 mg weekly × 3 weeks
Case-11	Physic education trainer	Biopsy embedded paraffin	Not done	Not done	*Leishmania* amastigotes (kinetoplast) in macrophages	Positive	Positive	Negative	No identified	Liposomal Amphotericin B 150 mg days 1–3 followed by 200 mg biweekly for three weeks
Case-14	Art Instructor	Scraping	Positive	Negative	Not done	Positive	Not done	Negative	No identified	Glucantime (20 mg/Kg/day) for 28 days

*This treatment was recommended to the patient, who returned to his country of origin

In 2 out of 5 positive cases, the infecting parasite species were identified: Case 1 was confirmed as *L. braziliensis,* based on restrictions of the hsp70-PCR protocol, whereas Case 2 was confirmed *L. mexicana* complex infection, according to PCR-N/RFLP products. *Leishmania* species was not identified in other 3 samples.

Concerning treatment, Case 1 was recommended to use IM Glucantime injections (20 mg/Kg/day for 20 days) on the return to his residence country, which occurred promptly after the diagnosis. Cases 2, 4, and 11 received Amphotericin and liposomal Amphotericin B, according to their availability, in different therapeutic schemes (Table 2). Case 14 completed a cycle of Glucantime, previously indicated at the country of exposure (20 mg/Kg/day for 28 days) (Table 2). All cases went through clinical re-evaluation (except for Case 1, who returned to his country of origin), the cure of initial lesions with no appearance of new ones was achieved.

Most of the patients (11/16) had a final diagnosis other than leishmaniasis (psoriasis, T-cell lymphoma, typhoid fever, leprosy, hyper ascaridiasis, or lympho-monocitary vasculitis); they receive treatment, attention and follow up accordingly.

## Discussion

Cutaneous leishmaniasis is highly prevalent in the American continent, where almost 67,000 cases per year have been reported [3]. The geographical position of Cuba favors travelers and possible subclinical patients from several leishmaniasis endemic countries, including Colombia (17,420 cases/year), Costa Rica (1249 cases/year), Bolivia (2647 cases/year), and Venezuela (2480 cases/year) [3]. As the GeoSentinel experience informed, most of the CL cases diagnosed in travellers visiting Central America and Mexico (1996–2010) (3.3%) were infected in Costa Rica and Belize [20]. In this regard, Barry et al. (2014) confirmed leishmaniasis in 4 Cuban immigrants travelling through this area, including the Darien jungle in Panama [21]. This indicates the necessity of enhancing the knowledge about the risks to get *Leishmania* parasites, and the need to be advised on measures to prevent insect bites in risky areas.

Recent reports of numerous imported leishmaniasis cases from the New World countries have been made in the Netherlands, Italy, and Australia [22–24]. While the burden of imported cases in non-endemic Caribbean countries is not available, the actual positive cases in Cuba it was expectable to be higher than our confirmed rates (5/16 cases during our examined ten years) or increase in the future, due to the expansion of travelers and human movement. This is the only study that describes clinical, epidemiological and diagnostic approaches used to elucidate a series of probable imported cases of leishmaniasis in Cuba during a 10 year’s period. This information not only shows how those cases were diagnosed and cured, but calls the attention of physicians and future travelers about the actual situation of a parasitic disease that is almost unknown in Cuba.

The parasitological analysis of the sample by direct microscopy, histopathology or culture, remains the gold standard in leishmaniasis diagnosis because of its high specificity [25]. These methods are also less costly and the most available in endemic settings. However, several factors associated with the lacking of expertise to identify the parasite, the variety of clinical samples that can be analyzed, and the high probability of contamination in culture, point to the need for the implementation of more sensitive and less subjective approaches for diagnosis in travel clinics and reference centers in non-endemic areas [26]. In that sense, molecular diagnosis has become a potential strategy to provide early detection and consequently the fast treatment implementation, as well as for species characterization, assessment of treatment efficacy and monitoring of relapses [27].

In the series analyzed, the results of parasitological approaches were critical for a final diagnosis, considering that at the moment in which most of these patients were attended at the institute; molecular methods were on evaluation in the lab. At the same time, some difficulties might be present, mainly associated to a low expertise in the sample taking, and the fact that microscopical examination for *Leishmania* is rarely made in Cuba. However, as a result of the application of conventional assays available, the first positive cases (Cases 1, 2, 4) were promptly diagnosed and treated for CL.

The infecting parasite species could not be always confirmed. In Case 1, *L. braziliensis* was firstly identified by using a monoclonal antibody-based approach with the collaboration of PECET (Programa de Estudio y Control de Enfermedades Tropicales, Universidad de Antioquia, Medellin, Colombia). This was corroborated in our lab years later by using PCR-*hsp*70/RFLP [13]. The species of *L. mexicana* complex was identified in Case 2 via PCR-N/RFLP (through *Bsa*JI and *Hind*II restrictions) [14, 15]; however, this protocol can’t differentiate the individual species within the complex. Case 4 had a weak amplification and was insufficient for RFLP, probably due to a low quantity of the remaining DNA sample or a failure during the nucleic acid extraction process. Case 11 species identification was not conducted, because of no amplification after PCR-N (likely due to the amplicon size [18], as it occurred in Case 14). The superiority of molecular detection of parasite DNA and identification of *Leishmani*a spp. in clinical samples have been a remarkable approach to diagnose this parasitic disease in our conditions, as the risk of low sensitivity or subjective analysis is diminished. The molecular assays, combined with the parasitological methods, made the algorithm used for diagnosis till the present day [17], comprehensive and more efficient by the conjunction of more sensitive and specific protocols for *Leishmania* species detection and identification [11, 14, 15].

Although no autochthonous cases of leishmaniasis have been reported in Cuba, a potential risk of transmission cannot be totally disregarded since several species of *Lutzomyia* were described in the country. Among them, *L. orestes* is widely distributed on the island and even when is usually found in caves where a possible contact with humans is reduced, it has been found able of biting and blood-feeding from humans [28].

The surveillance and follow up of imported cases of infectious parasitic diseases, e.g. leishmaniasis and others transmitted by vectors, among others, must be a task for medical and laboratory personnel, as it must be a duty for counselors of travel clinics and humanitarian organizations to advise possible travellers to endemic areas how to avoid parasitic infections.

## Conclusions

This work demonstrates that an algorithm combining parasitological and molecular methods is useful for the diagnosis of imported leishmaniasis in Cuba. As a non-endemic country, the use of molecular approaches is preferable, because they are highly sensitive and less subjective, allowing possible the identification of the infecting species. Trained personnel should be ready to offer advice, proper attention and differential diagnosis. Information and research like this report are relevant, given the increased travel or human movement.

## Abbreviations

18S rRNA: 18S fragment from the ribosomal ribonucleic acid
*hsp*20: gene coding for the heat shock protein of 20 kDa
*hsp*70: gene coding for the heat shock protein of 70 kDa
IPK: Institute of Tropical Medicine Pedro Kourí
ITS: ribosomal internal transcribed spacer
kDNA: kinetoplast DNA
PCR: Polymerase chain reaction
